# A case of adrenomyeloneuropathy caused by a novel point mutation in the *ABCD1* gene and functional verification

**DOI:** 10.3389/fgene.2024.1421122

**Published:** 2024-09-27

**Authors:** Xiaoxue Shi, Xuelin Qi, Jinhua Zheng, Jianjun Ma, Dongsheng Li

**Affiliations:** ^1^ Department of Neurology, Henan Provincial People’s Hospital, Zhengzhou, China; ^2^ Department of Neurology, Zhengzhou University People’s Hospital, Zhengzhou, China; ^3^ Department of Neurology, Henan University People’s Hospital, Zhengzhou, China

**Keywords:** *ABCD1* gene, adrenomyeloneuropathy (AMN), point mutation, genetic analysis, functional verification

## Abstract

Adrenoleukodystrophy is a rare neurogenetic disease, and adrenomyeloneuropathy is the most common phenotype in adults. The clinical data of a patient with adrenoleukodystrophy and spinal-peripheral neuropathy caused by a novel point mutation in exon 4 of the *ABCD1* gene (c.1256T > G (p.Val419Gly)) were retrospectively analyzed. Furthermore, we constructed wild-type and mutant vectors of the *ABCD1* (NM0000334) gene to validate the effect of this mutation on the expression of the *ABCD1* gene and protein and to explore the mechanism of X-linked adrenoleukodystrophy occurrence and development to identify therapeutic targets for clinical treatment.

## 1 Introduction

X-linked adrenoleukodystrophy (X-ALD) is a hereditary peroxisome metabolic disease caused by a mutation in the *ABCD1* gene. This gene is located at Xq28 and encodes adrenoleukodystrophy protein (ALDP), which promotes the transport of very-long-chain fatty acids (VLCFAs) to peroxisomes for degradation (Wang et al., 2015; Engelen et al., 2014). The neurological symptoms of adrenomyeloneuropathy (AMN) generally occur from the ages of 20–50 and manifest as chronic progressive paraparesis accompanied by sensory and sphincter disturbances (Griffin et al., 1977). Here, we confirmed the diagnosis of AMN in a 31-year-old adult male patient and identified a novel *ABCD1* gene mutation (p.Val419Gly) using whole-exome sequencing.

According to the X-ALD database (Richards et al., 2015), the detected *ABCD1* mutation was a novel mutation. Therefore, we constructed wild-type (WT) and mutant (MUT) vectors of the *ABCD1* (NM0000334) gene to verify the influence of this mutation on the expression of the ABCD1 gene and protein, explore the mechanism of X-ALD occurrence and development and provide therapeutic targets for use in the clinic.

## 2 Methods

### 2.1 Clinical presentation

**FIGURE 1:**
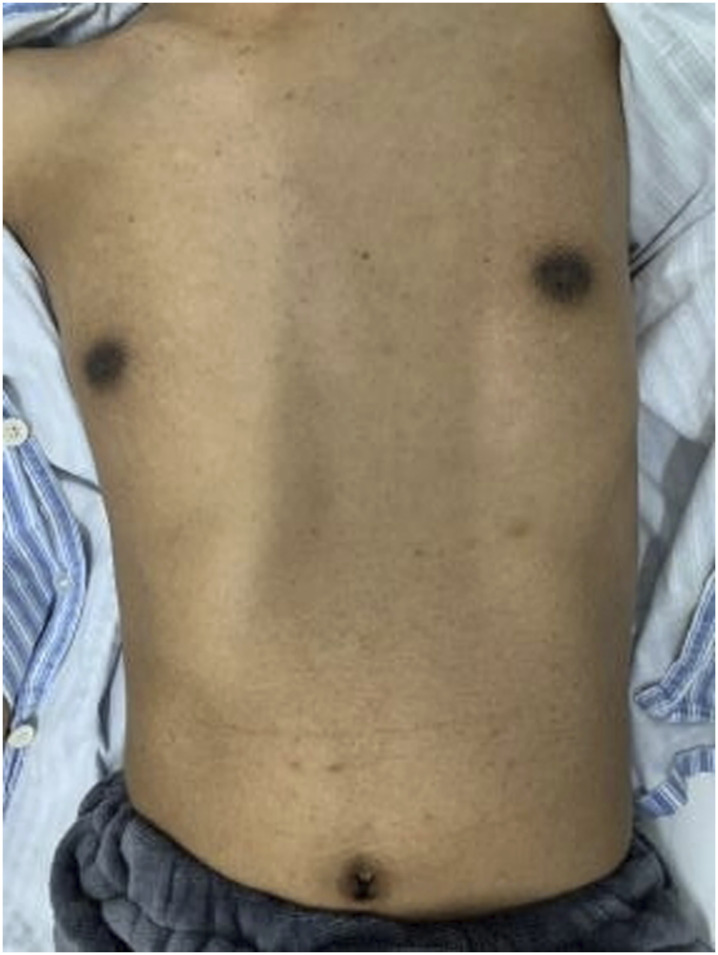
The patient exhibited black skin and pigmentation of the areola and navel.

The patient was a 31-year-old adult male who presented with progressive deterioration of bilateral lower extremity weakness and numbness with dysuria for 1 year. The patient exhibited black skin and pigmentation of the areola and navel (Figure 1). The muscle strength of his lower limbs was Grade IV, muscle tension was increased, the tendon reflexes of the four limbs were active, and the pathological reflexes were positive. The Romberg sign was positive, with decreased superficial sensation below the knee. Brain magnetic resonance imaging (MRI) performed at the local hospital showed no abnormalities.

The patient’s blood sodium level was 124 mmol/L (reference range, 137–147), adrenocorticotrophic hormone (ACTH) level at 8 am was >1,250 μg/dL (reference range, 12–46), cortisol level at 8 am was 5.02 μg/dL (reference range, 6.7–22.6), renin activity (standing position) was 18.4 ng/ml/h (reference range, 0.1–6.56), angiotensinogen II level (lying position) was 73.3 pg/mL (reference range, 25–60), and aldosterone level (upright position) was 57.2 pg/mL (reference range, 70–300). VLCFA analysis revealed increased serum C26 levels and C26/C22 and C24/C22 ratios (Table 1). Plain and enhanced MR images of the thoracolumbar spinal cord were obtained. The lumbar spinal cord showed no obvious abnormalities, and the thoracic spinal cord was straight and thin. Electrophysiological examination revealed that the H-reflex of both lower limbs showed changes in central inhibition, and the deep sensation and pyramidal tract conduction functions, mainly involving both lower limbs, were abnormal.

**TABLE 1:** Very long chain fatty acid level (nmol/L) in the patient and reference values.

Test	Results, nmol/mL	Reference values, nmol/mL
C22:0	37.3	≤96.3
C24:0	59.5	≤91.4
C26:0	3.22	≤1.30
C24/C22	1.60	≤1.39
C26/C22	0.086	≤0.023

### 2.2 Ethics statement

Written informed consent was obtained from the proband, his parent and his son before performing genetic analysis. Institutional Review Board (IRB) approval was not required since genetic analysis is an essential and routine diagnostic method for patients with X-ALD. Failure to perform genetic analysis can lead to inadequate diagnosis and render genetic counseling impossible.

## 3 Materials and methods

### 3.1 Genetic analysis

The genetic analysis method consists of three main steps: mutation screening using high-throughput sequencing, gene data analysis using bioinformatics and clinical information analysis, and verification of suspected pathogenic mutations using Sanger sequencing. 1. Mutation screening: Whole-exome sequencing was performed, and the samples were subsequently sequenced using the IDT xGen Exome Research Panel v2.0 whole-exome capture chip. 2. Gene data analysis: System analysis and screening was performed using the genetic disease precise diagnosis cloud platform, which integrates molecular biology annotation, biology, genetics and clinical feature analysis. Hundreds of thousands of gene variations were assessed using databases of pathogenic mutations, the normal human genome, and clinical characteristics of 4,000 known genetic diseases and a gene data analysis algorithm; the variations were classified using the three-factor classification system and the American College of Medical Genetics and Genomics (ACMG) gene variation classification system. 3. Verification of suspected pathogenic mutations: After polymerase chain reaction (PCR), the target sequence was verified by Sanger sequencing with an ABI 3730 sequencer, and the results were verified by sequence analysis software.

**FIGURE 2:**
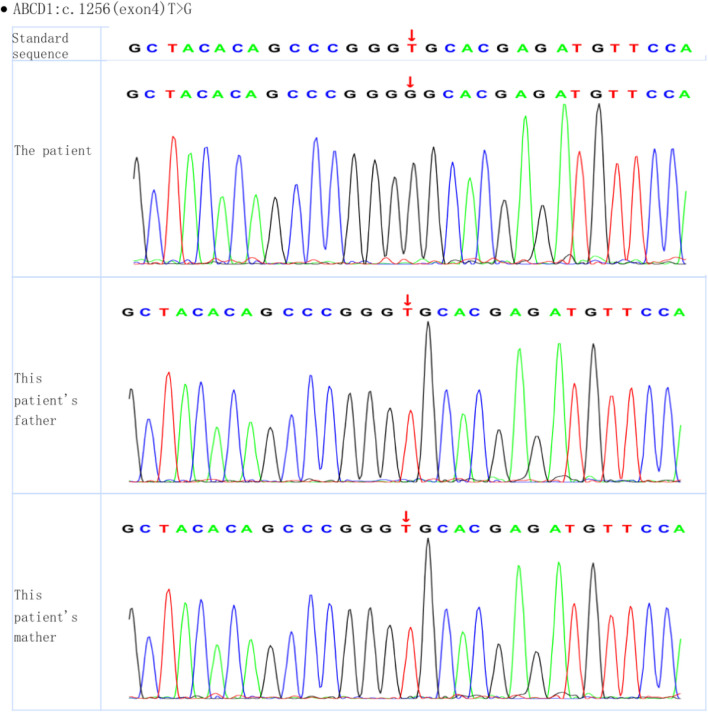
Whole-exome sequencing of the proband and his parents.

**FIGURE 3:**
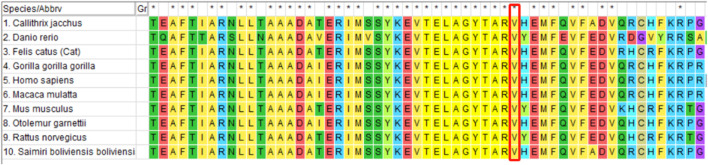
Amino acid conservation analysis (red box): the 419Val locus is highly conserved in different species.

Whole-exome sequencing of the proband revealed the presence of a mutation generating a stop codon in exon 4 (c.1256T > G (p.Val419Gly)). Sanger sequencing confirmed that this mutation had not been reported previously (according to the ALD mutation database (Kemp et al., 2001)) (Figure 2). Amino acid conservation analysis: After querying the NCBI genomic database and using T-coffee software for analysis, the 419Val locus was found to be highly evolutionarily conserved across species (Figure 3). The homozygous *de novo* mutation in the proband was consistent with the pathogenesis of autosomal dominant (AD) inheritance, and the cosegregation of phenotype and genotype in the proband and his family members was consistent. The variation met the PM2_Supporting, PM6, PP3 and PP4 criteria of the ACMG guidelines (Richards et al., 2015). These results suggested that the harmfulness of the *ABCD1* gene mutation may be related to the phenotype of the patient.

### 3.2 Functional verification

For cell culture, 293T cells were purchased from Beina Biology (BNCC353535) and cultured in a 37°C 5% CO2 incubator (BPN-80CW, Shanghai Yiheng Science Instrument Co., Ltd.). For cell transfection, when the cell density reached 70%, the transfection medium was prepared; the medium of the cells was replaced with serum-free medium, and 5 μL of Lipofectamine 3,000 (L3000015, Invitrogen) and 2.5 µg of WT were added. The mutant ABCD1 plasmid has the same transfection conditions as the wild-type ABCD1 plasmid. The mRNA was extracted by CW0581M (CWBIO) for qRT‒PCR detection, and the concentration and purity of the mRNA were determined. For western blotting, total protein was extracted by homogenizing animal tumors with a tissue grinder after RIPA lysis buffer was added, and the proteins were visualized with a supersensitive chemiluminescence imaging system. The mutant *ABCD1* plasmid has the same transfection conditions as the wild-type ABCD1 plasmid.

GraphPad Prism 9.0 software was used for graphic rendering and statistical analysis. All experiments were repeated 3 times, and the quantitative results are expressed as the mean ± standard deviation. Single-factor analysis of variance (ANOVA) was used for quantitative comparisons among groups, and the S-N-K method was used for pairwise comparisons. *P* < 0.05 indicated a significant difference.

**FIGURE 4:**
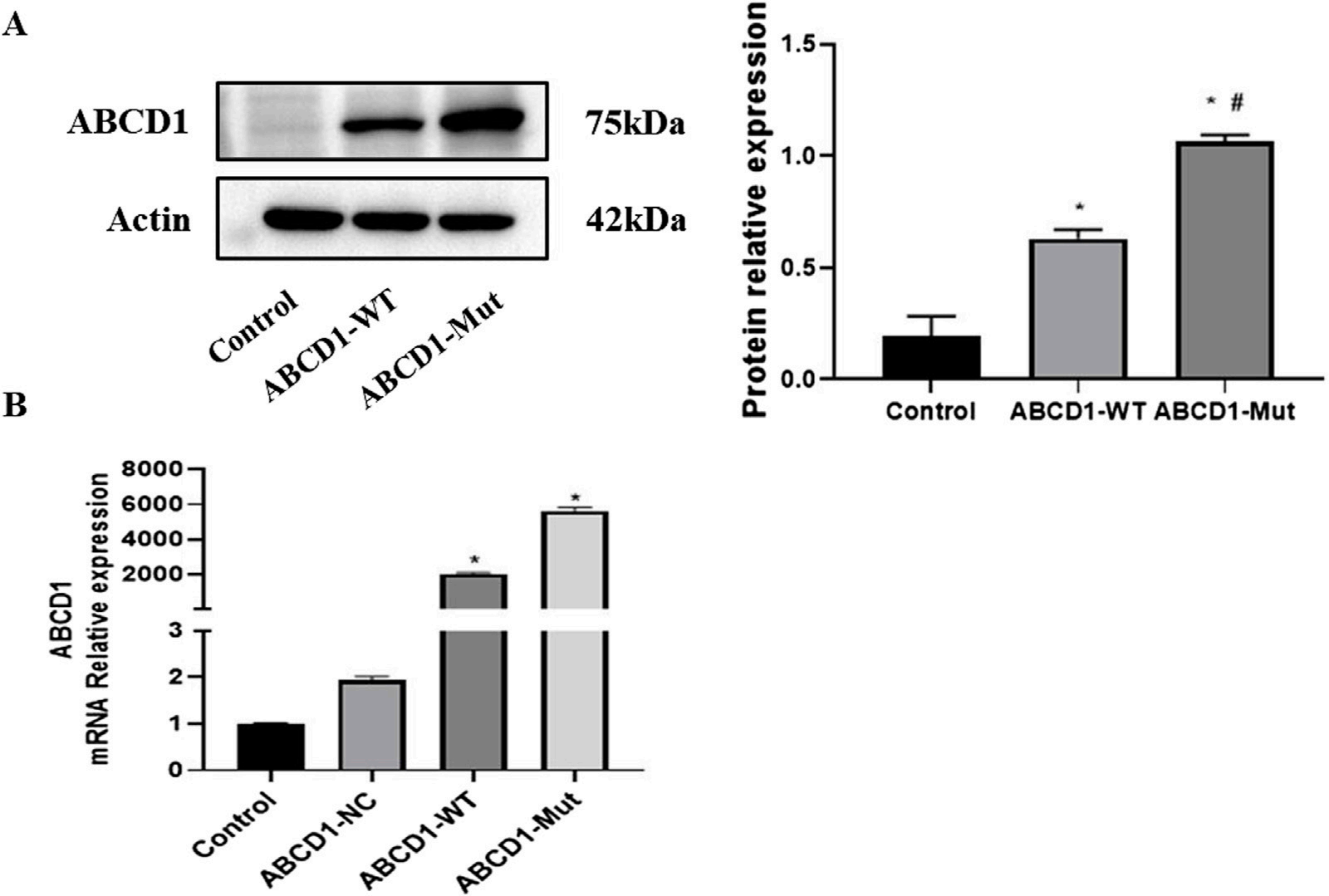
**(A)** The result of protein relative expression; **(B)** The result of mRNA relative expression (The control group is cells without plasmid, and the NC group is cells without empty plasmid carrying the target sequence.).

The Western blotting and PCR results showed that the protein and mRNA levels of *ABCD1*-WT and ABCD1-MUT were significantly greater than those in the control and NC groups (Figure 4).

## 4 Discussion

AMN is one of the main phenotypes of ALD, and neurological symptoms usually occur at age 20–50, resulting in progressive paraplegia, sphincter disorder, sexual dysfunction and impaired adrenal cortex function (Budka et al., 1976). Smith KD et al. discussed the lack of correlation between the genotype and phenotype in X-ALD patients (Smith et al., 1999). First, even patients with a large deletion or frameshift mutation that leads to complete deletion of the protein will exhibit a mild AMN phenotype. Second, even if X-ALD occurs as a hereditary disease in multiple family members, the clinical phenotypes of family members can be different, which is confirmed by the phenotypes of the clinical spectrum of X-ALD that have been described (Dong et al., 2022). Third, even in identical twins, different clinical phenotypes were observed (Korenke et al., 1996). However, it cannot be ruled out that specific mutations may lead to residual activity of the *ABCD1* protein, which may affect the severity of the phenotype (O’Neill et al., 2001). G Unterrainer et al. pointed out that mutated ALDP competed with normal ALDP to integrate into a limited number of sites in the peroxisome membrane, and concluded that an increase in the number of ALDP mutations led to a decrease in peroxisome β-oxidation and the accumulation of long-chain fatty acids (Unterrainer et al., 2000). Similarly, the increased expression of mutant ABCD1 in our study is likely to be parallel to the accumulation of long-chain fatty acids, thus reflecting the severity of the disease.

We identified a new point mutation in exon 4 of the *ABCD1* gene. Gene sequencing revealed that the base T was replaced by G at position 1,256, resulting in a missense mutation. Based on clinical symptoms, positive neurological signs, family history, and imaging and biochemical characteristics, we believe that this mutation is a pathogenic variation. Therefore, we used cell bioengineering technology to transfect the target gene into 293T cells, constructed wild-type and mutant vectors of the *ABCD1* gene, and analyzed the molecular weight and intracellular expression level of the corresponding mRNAs and proteins after transcription and translation. A lack of obvious differences would indicate that the mutation is not a pathogenic variation. However, as shown in the figure, there were obvious differences in both the molecular weight and expression level of the protein and the expression level of the related mRNA after transcription.

The ABCD1 protein is encoded by 10 exons; exons 1 and 2 encode transmembrane domains (TMDs) responsible for substrate specificity, while exons 5–10 encode ATP-binding domains (Dohr et al., 2023). Our variant was located in exon 4, and no nonsense-mediated activation of mRNA attenuation was observed. The translation of *ABCD1* with the c.1256T > G mutation will cause a protein with a p. Val419Gly amino acid change. To date, only 3% of identified missense/nonsense mutations have been located in exon 4, and only 1% of them are related to a mild AMN phenotype (Stenson et al., 2003).

Hormone therapy is an established treatment for adrenal insufficiency (Raymond et al., 2007). In some patients with adrenal insufficiency, glucocorticoids and mineralocorticoids may be needed to maintain the water-electrolyte balance, but they do not significantly affect neurological signs or symptoms. Nutritional interventions, such as a low-fat diet and Lorenzo oil, can reduce VLCFA levels (Aubourg et al., 1993). At present, hematopoietic stem cell transplantation (HSCT) is the only successful treatment option for early brain ALD. In contrast, bone marrow transplantation is not an optimal choice for seriously ill patients because of its high risk and uncertain effects (Gordon et al., 2014). Gene therapy using autologous hematopoietic stem cells rather than allogeneic hematopoietic stem cell transplantation has also made progress in recent years. Patients with adrenal leukodystrophy were infused with a lentiviral vector containing *ABCD1* complementary DNA (cDNA) transduced with autologous CD34^+^ hematopoietic stem cells *in vitro*, and the results showed that this approach could effectively prevent the progression of brain demyelination; this effect was equivalent to that obtained by allogeneic HSCT (Cartier et al., 2009). In animal experiments, gene delivery of intrathecal recombinant adeno-associated virus (AAV) by an osmotic pump led to extensive gene expression in the central nervous system and a significant decrease in the level of VLCFAs in the spinal cord (Gong et al., 2019).

In summary, the selected treatment methods for ALD are limited, and some methods rely on early diagnosis and intervention. Early diagnosis is key to improving the morbidity and mortality of ALD patients. Therefore, gene sequencing analysis may become an important tool for neonatal screening and genetic counseling. At present, adrenal leukodystrophy is included in neonatal screening for diseases in some states in the United States.

## 5 Conclusion

In this case, we report a patient with peripheral neuropathy AMN, which was finally diagnosed after a year when the neurological presentation worsened. A novel *ABCD1* X-ALD mutation (c.1256T > G (p. Val419Gly) was identified, and its pathogenicity was successfully confirmed by bioengineering.

## Data Availability

The datasets presented in this article are not readily available because of ethical and privacy restrictions. Requests to access the datasets should be directed to the corresponding authors.
